# The efficacy of zinc finger antiviral protein against hepatitis B virus transcription and replication in tansgenic mouse model

**DOI:** 10.1186/s12985-015-0245-0

**Published:** 2015-02-13

**Authors:** En-Qiang Chen, Jie Dai, Lang Bai, Hong Tang

**Affiliations:** Center of Infectious Diseases, West China Hospital of Sichuan University, Chengdu, 610041 China; Division of Infectious Diseases, State Key Laboratory of Biotherapy, Sichuan University, Chengdu, 610041 China

**Keywords:** Zinc-finger antiviral protein, Hepatitis B virus, Transgenic mouse, Transcription and replication

## Abstract

**Aim:**

The zinc finger antiviral protein (ZAP) is a mammalian host restriction factor, and it could inhibit HBV RNA synthesis in vitro experiments. However, the role of ZAP against HBV in vivo environment is unclear. This study aimed to investigate whether ZAP could act against HBV transcription and replication in ZAP tansgenic mouse model.

**Methods:**

HBV-replication-competent plasmid pHBV4.1 was transferred to ZAP transgenic ICR mouse via the tail vein using a hydrodynamic in vivo transfection procedure, and ICR mouse were used as controls. HBV RNA and HBV DNA replication intermediates in the liver were respectively analyzed by Northern blotting and Southern blotting. The expression of hepatitis B surface antigen (HBsAg) and hepatitis B core antigen (HBcAg) in the liver tissue was detected by immunohistochemical staining.

**Results:**

As compared to ICR control mouse, the levels of 3.5 kb mRNA in ZAP transgenic mouse were decreased by 8.4%; while the level of HBV DNA replication intermediates was decreased by 82%. In addition, the expression of HBsAg and HBcAg in ZAP transgenic mouse liver were both significantly less than that of ICR control mouse.

**Conclusions:**

Our findings suggest that ZAP could inhibit HBV replication in vivo in mice, which offers a new target for anti-HBV drug development.

## Background

Hepatitis B virus (HBV) infection is a serious global public health problem, and approximately two billion people who have been infected worldwide. Of them, there are more than 350 million who are chronic carriers of HBV [1,2]. Sufficient evidences have showed that the level of serum HBV DNA is a strong predictor of HBV-related complications [3]. And how to effectively control and even eliminate virus replication has been concerned increasingly by clinicians [4,5].

In past decades, five oral nucleos(t)ide analogues (NAs) are approved for the treatment of chronic HBV infection [6-8], which decrease virus production by inhibiting the HBV DNA polymerase. However, because of the persistence of HBV covalently closed circular (cccDNA) in liver, current NAs treatment rarely eradicate HBV infection, and most patients require long-term NAs administration. Recently, with the extension of duration of treatment, the issues of drug resistance, viral relapse and particularly suboptimal response to current antiviral agents are increasingly evident [9-12], and how to manage those patients after initial therapy failure has become the primary concern for clinicians [5]. So the development of the effective drugs that target different stages of life cycle of HBV is still needed.

The zinc finger antiviral protein (ZAP) is a mammalian host restriction factor. Though ZAP itself does not have RNase activity, it could bind to viral RNA specific sequences and recruite the exosome to degrade the target RNA substrate [13]. For example, ZAP can specially inhibit the replication of murine leukemia virus (MLV), xenotropic murine leukemia virus-related virus (XMRV), sindbis virus (SIN) [14], human immunodeficiency virus type 1(HIV-1), ebola virus, marburg virus [15-17] and so on. Indeed, the inhibition activity of ZAP is not just restricted to positive-strand RNA viruses, and retroviruses are also targets of ZAP. One recent in vitro study has shown that ZAP also plays a role in the innate control of HBV replication, through down-regulation of viral RNA [18]. However, it is still unclear whether in vivo and in vitro environments have different effects on ZAP against HBV, because ZAP does not induce a universal antiviral state and some viruses could replicate normally in ZAP-expressing cells [19]. To clarify the effect of ZAP against HBV in vivo environment, present study was designed to evaluate the efficacy of ZAP against HBV transcription and replication in ZAP tansgenic mouse model.

## Materials and methods

### Plasmids

The plasmid pHBV4.1(ayw subtype), which contains 1.3 copies of the HBV genome, includes the viral sequence from nucleotide coordinates 1072 to 3182 plus 1 to 1990. This construct has been proven to initiate replication of HBV efficiently after being transfected into human hepatoma HepG2 cell lines [20,21]. The plasmid pEFmZAPmyc was a ZAP expression plasmid, which was constructed by integration of mZAPmyc fragment into the plasmid pEF/myc/nuc/GFP (Invitrogen).

### The ZAP transgenic mouse and HBV replication mouse model

All mice used in this study were breeded at specific-pathogen-free (SPF) level, weighing 22-24 g. The ZAP transgenic mouse and ICR mouse were provided by Center for Infections and Immunity, Institute of Biophysics, Chinese Academy of Sciences, Beijing, China. For construction of the transgenic mouse, the plasmid pEFmZAPmyc was injected into the fertilized egg nucleus of ICR mouse by microinjection, transferred into uterine tube of the fake pregnant female mouse and identified successfully.

In order to establish the mouse model for HBV replication, 10 μg of pHBV4.1 plasmids diluted in phosphate-buffered saline (PBS) was injected into the mouse tail vein in a volume of 10% of the body weight (v/w) within 5-8 seconds (hydrodynamic in vivo transfection) [22]. ZAP transgenic mouse transfected pHBV4.1 was termed the ZAP-ICR/HBV mouse as experimental group, and ICR mouse transfected pHBV4.1 was termed ICR/HBV mouse as control group. All mice were sacrificed after 3 days of pHBV4.1 injection and liver tissues were collected.

### Analysis of HBV RNA in mice liver by Northern blotting

Frozen mice liver tissue mechanically pulverized in liquid nitrogen were processed for RNA extraction using Trizol reagent (Invitrogen, USA) following the manufacturer’s instructions. The 30 μg total HBV RNA was analysed by Northern blotting hybridization and probed for both digoxigenin-labeled (Roche, Swiss) GAPDH and HBV, as previously described [23]. After electrophoresis, transfer, UV cross-linking, prehybridization, hybridization, washing and chromogenic reaction, the membranes were exposed using Kodak RX films (Eastman Kodak Co, USA) for 0.5-1 hour at 37°C.

### Analysis of HBV DNA replication intermediates in mice liver by Southern blotting

Frozen liver tissues were mechanically pulverized in liquid nitrogen and HBV DNA replication intermediates were isolated from 120 μg liver tissue powders as described previously. All isolated of HBV DNA replication intermediates were diluted to 30 μL with TE buffer and then were analyzed by DNA Southern blotting as previously described. Membranes were hybridized with digoxigenin-labeled (Roche Applied Science) HBV ayw genomic DNA to detect HBV sequences. The levels of HBV DNA replication intermediates were calculated by the Quantity One software according to manufacturer’s instructions (Bio-Rad, USA).

### Detection of HBsAg and HBcAg in mice liver by immunohistochemical staining

The number and location of cells in the liver that expressed HBsAg or HBcAg were assessed by immunohistochemical staining. Liver tissue fixed in formalin was embedded with paraffin and sectioned (3 mm thick). The paraffin-embedded sections in PBS were treated for 15 min at 37°C with 30 g/L hydrogen peroxide, and washed with PBS. After the sections were blocked with normal goat serum for 20 min at 37°C, mouse anti-HBsAg (Thermol) or rabbit anti-HBc (NEOMARKERS) primary antibody was applied at 1:100 or 1:150 dilutions for 60 min at 37°C, or overnight at 4°C, respectively. After washing with PBS, the secondary antiserum consisting of Polymer-HRP Anti-Rabbit (Zhongshan, Beijing) was applied at 1:350 dilutions for 50 min at 37°C, according to the manufacturer’s instructions. The antibody-coated sections were washed with PBS, stained with 3′, 3′-diaminobenzidine tetrahydrochloride (DAB), and counterstained with hematoxylin, before being mounted.

## Results

### The effect of ZAP on HBV transcription and replication

**Figure 1:**
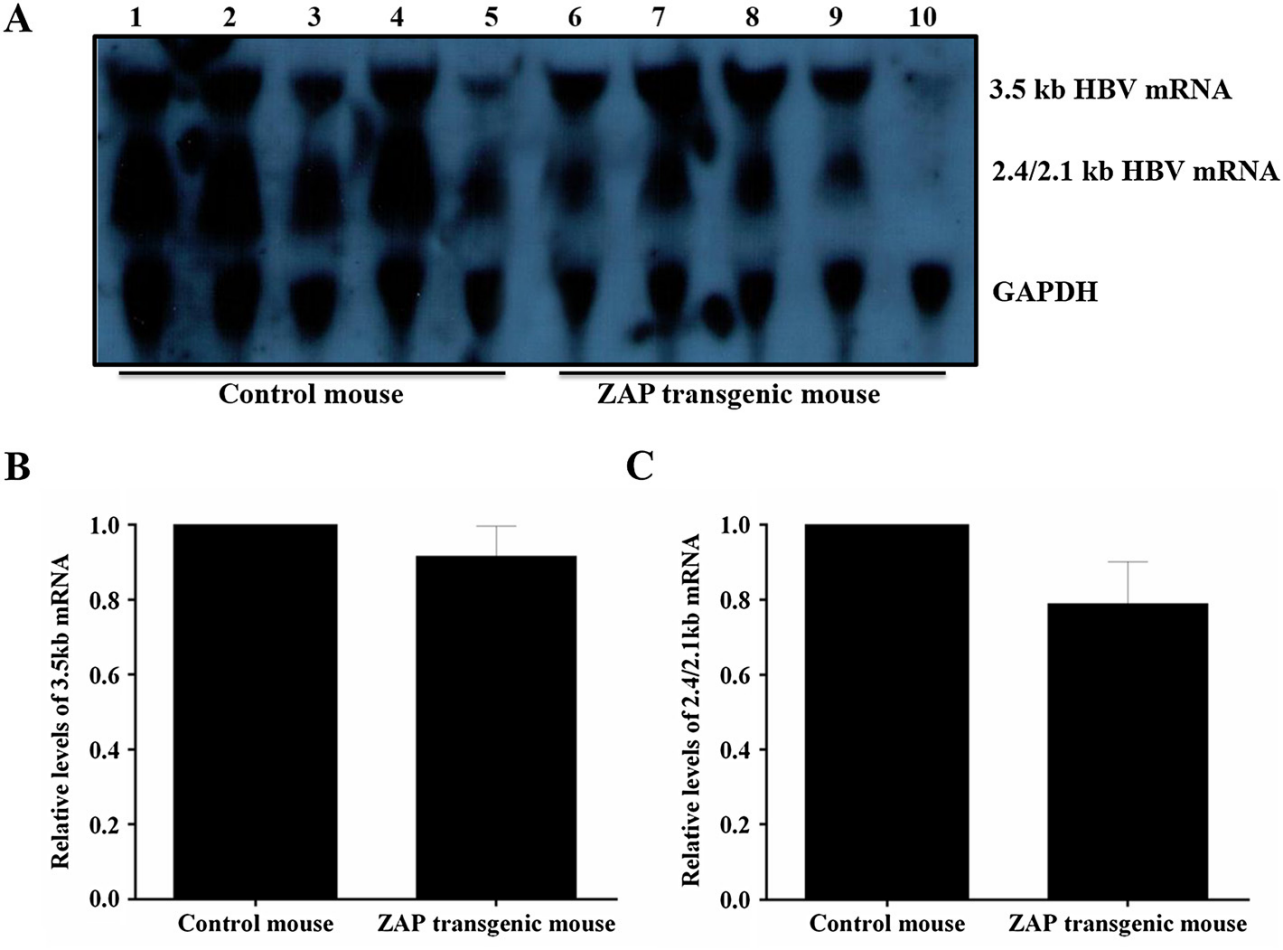
**The transcription levels of HBV RNA in mouse liver tissue. (A)**: Lanes 1-5 for ICR control mouse and lanes 6-10 for ZAP transgenic mouse. **(B)**: Quantitative analysis results of the 3.5-kb HBV mRNA. **(C)**: Quantitative analysis results of the 2.4/2.1-kb HBV mRNA. The mean value of ICR control mouse was defined as 1, and GAPDH was used as internal control.

In present study, the 3.5 kb mRNA and 2.4/2.1 kb mRNA of HBV were detected in both ZAP transgenic mouse and control mouse (Figure 1A). As compared to control mice, the levels of 3.5 kb mRNA in ZAP transgenic mouse were decreased by 8.4% (Figure 1B); while the levels of 2.4/2.1 kb mRNA in ZAP transgenic mouse were decreased by 21.1% (Figure 1C).

**Figure 2:**
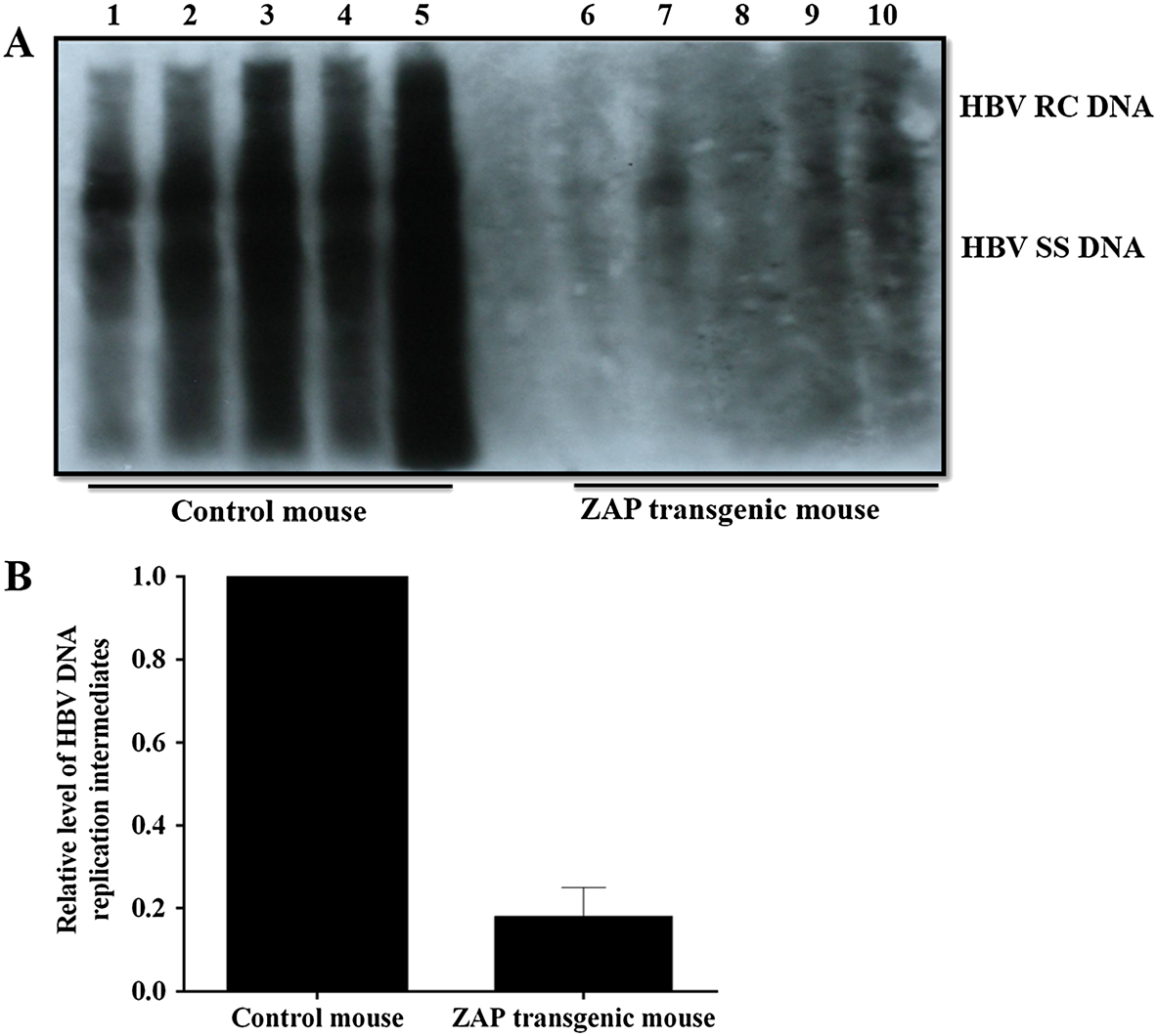
**The replication levels of HBV DNA in mouse liver tissue. (A)**: Lanes 1-5 for ICR mice and lanes 6-10 for ZAP transgenic mouse. **(B)**: Quantitative analysis results of the relative intensity of HBV DNA replication intermediates. The mean value of ICR control mouse was set to 1.

As shown in Figure 2, either the levels of HBV RC DNA or HBV SS DNA in ZAP transgenic mouse were decreased significantly than that in control mouse (Figure 2A). As compared to control mouse, the levels of HBV DNA replicative intermediate in ZAP transgenic mouse were dereased by 82% (Figure 2B).

### The effect of ZAP on HBsAg and HBcAg expression

**Figure 3:**
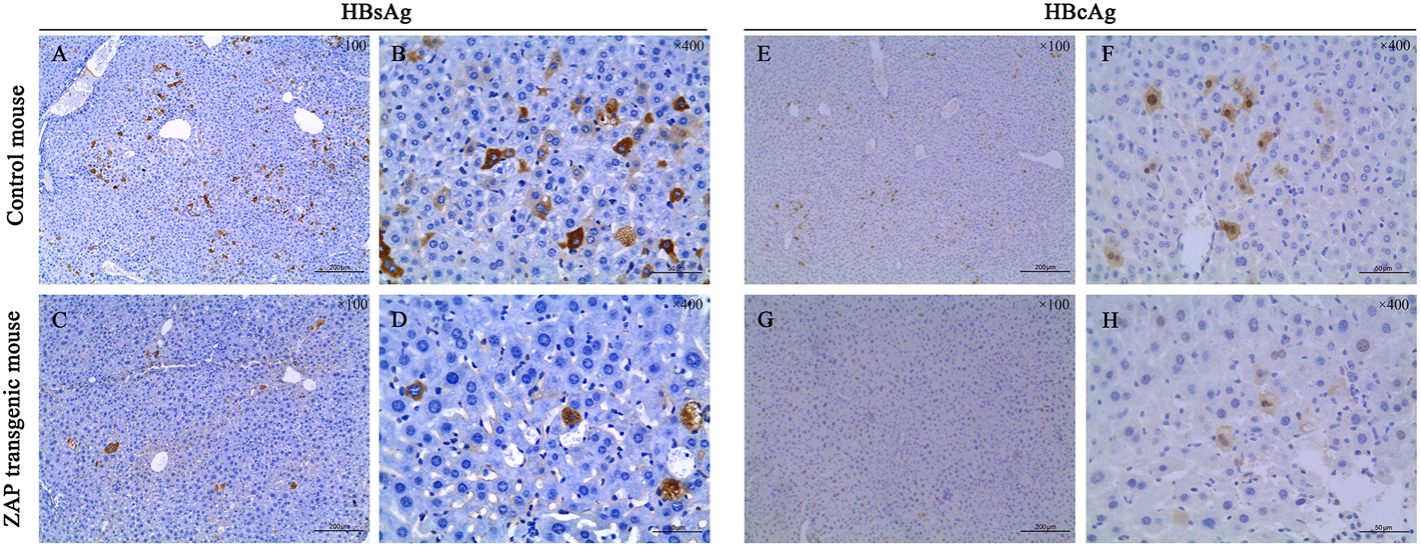
**Immunohistochemical staining of HBsAg (A-D) and HBcAg (E-H) in liver tissue sections.**

As shown in Figure 3 (A-D), the staining of HBsAg was presented as brown in the cytoplasm. There were only very few HBsAg-positive cells in liver tissue of ZAP transgenic mouse(C and D); while HBsAg-positive hepatocytes were abundant in liver tissue of control mouse(A and B).

As shown in Figure 3 (E-H), the staining of HBcAg was presented as brown in the nuclei and cytoplasm. The number of HBcAg-positive cells in ZAP transgenic mouse(G and H) was significantly less than that in control mouse(E and F), and the intensity of HBcAg-staining in ZAP transgenic mouse was also weaker than that in control mouse.

## Discussion

Recently, it has been reported that ZAP could inhibit a variety of viruses replication, through interaction with the ZAP-responsive elements (ZRE) in viral RNA and recruiting the exosome to degrade RNA substrate [17,24]. In spite of these achievements made, there are still inadequacies in the full biological characteristics of ZAP. Because all studies about the antiviral effect of ZAP are at cell level in vitro, and there is no report about ZAP against HBV in vivo environment. To our knowledge, either the virol/host proteins or their interactions in regulating HBV replication would be influenced by the types of cells and microenvironment change [21,25]. So, it is necessary and important to investigate the role of ZAP against HBV in vivo models. The major findings in ZAP transgenic mouse from present study are: (1) a slight decreasing of HBV RNA, (2) a significant decreasing of HBV DNA replication intermediates, (3) decreasing of viral proteins expression.

As we know, host ZAP could be naturally expressed in both ICR mouse and human body, but its expression level is very low, which restricts the research progress of ZAP against HBV to a certain degree. At present, there are two widely used methods to induce target protein expression in vivo, which include plasmid transfection and transgene expression. As compared to conventional plasmid transfection, transgene expression method is easy to obtain a stable expression of target genes. With kind assistance from Porf. Gao GX and his research team, who had huge achievements on ZAP discovery and biological functional study [24,26-28], we obtained a ZAP transgenic mouse (data unshown in present study). For simulating an in vivo environment with relative high ZAP expression and HBV replication, HBV expression plasmid pHBV4.1 was subsquently delivered to this ZAP transgenic mouse liver via tail vein injection. Previous study had found that the HBV DNA replication intermediates in this mouse model were abundant on day 3 and 4, and disappeared on day 10 [29], thus day 3 after transfection was a widely used timepoint to evaluate the change of HBV transcription and replication levels [30,31]. Based on the previous research experience, the role of host ZAP against HBV transcription and replication in present study was also evaluated at this timepoint.

It has been revealed that 3.5 kb mRNA contains all genetic information of HBV DNA and plays a very important role in the life cycle of HBV as the template of replication. So in present study, the level of 3.5 kb mRNA was used to reflect the transcriptional level of HBV in both ZAP transgenic mouse and control mouse. In the study reported by Prof. Mao and his colleague showed that overexpression of ZAP could efficiently reduce HBV RNA in cell cultures [18]. In present study, we also observed a decreasing of 3.5 kb HBV mRNA as well as 2.4/2.1 kb HBV mRNA in ZAP transgenic mosue in vivo. Though the extent of 3.5 kb HBV mRNA decline was not obvious in ZAP transgenic mosue, this finding was still consistent with previous reports [18], because both in vivo and in vitro experiments indicated a tendency of HBV RNA dropping. Though ZAP-responsive element was mapped in terminal redundant region (nt 1820–1918) of HBV pgRNA, ZAP expression didn’t alter the amount or stability of HBV RNA transcription templates, because the activities of HBV core as well as S1 and S2 promoters was not significantly affected by ZAP [18]. So there was an inference that ZAP-mediated HBV RNA reduction was not due to a transcriptional inhibition, but rather through accelerating HBV RNA decay [18]. Considering the less decreasing of 3.5 kb HBV mRNA in vivo than in vitro, we speculated the process of ZAP recruiting the RNA processing exosome to degrade HBV RNA may be influenced in ZAP transgenic mouse by some unknown factors, and the changes between in vitro and in vivo environment may be associated with it. And future studies are strongly required to confirm this speculation.

In preset study, the significant decline of HBV DNA replication intermediates in ZAP transgenic mouse was consistent with previous findings in vitro experiment. At present, it was well-known in vitro experiment that ZAP inhibited HBV replication through posttranscriptional down-regulation of viral pgRNA [18]. To our knowledge, many evidences had suggested that in vivo very subtle changes in HBV transcription may contribute to large alterations, either negative or positive, in viral replication [32]. So we believed that the significant decline of HBV DNA replication intermediates in ZAP transgenic mouse should be explained by above mentioned HBV RNA decay. Additionally, the expression of HBsAg and HBcAg were also inhibited in ZAP transgenic mouse in present study. We thought the significantly decline of HBV DNA replication intermediates should be the main cause of the inhibition of virol protein expression.

In summary, present study firstly investigate the effect of ZAP against HBV transcription and replication in vivo environment, and the results suggest that ZAP can also effectively inhibit HBV replication. This finding will offer a new potential target for anti-HBV drug development.

## References

[1] Liaw YF, Chu CM (2009). Hepatitis B virus infection. Lancet.

[2] Lok AS, McMahon BJ (2007). Chronic hepatitis B. Hepatology.

[3] Lin CL, Kao JH (2011). Recent advances in the treatment of chronic hepatitis B. Expert Opin Pharmacother.

[4] Zhang NP, Reijnders JG, Perquin M, Hansen BE, Janssen HL (2011). Frequency and clinical outcomes of flares related to nucleos(t)ide analogue therapy in patients with chronic hepatitis B. J Viral Hepat.

[5] Chen EQ, Tang H (2014). Optimization therapy for the treatment of chronic hepatitis B. World J Gastroenterol.

[6] Iloeje UH, Yang HI, Su J, Jen CL, You SL, Chen CJ (2006). Predicting cirrhosis risk based on the level of circulating hepatitis B viral load. Gastroenterology.

[7] Chen CJ, Yang HI, Su J, Jen CL, You SL, Lu SN (2006). Risk of hepatocellular carcinoma across a biological gradient of serum hepatitis B virus DNA level. JAMA.

[8] Vigano M, Lampertico P (2011). Antiviral drugs for HBV liver disease. Expert Opin Biol Ther.

[9] Shamliyan TA, MacDonald R, Shaukat A, Taylor BC, Yuan JM, Johnson JR (2009). Antiviral therapy for adults with chronic hepatitis B: a systematic review for a National Institutes of Health Consensus Development Conference. Ann Intern Med.

[10] Wang Z, Wu XL, Zeng WZ, Xu H, Zhang Y, Qin JP (2011). Lamivudine plus adefovir is a good option for chronic hepatitis B patients with viral relapse after cessation of lamivudine treatment. Virol J.

[11] Wang LC, Chen EQ, Cao J, Liu L, Wang JR, Lei BJ (2010). Combination of Lamivudine and adefovir therapy in HBeAg-positive chronic hepatitis B patients with poor response to adefovir monotherapy. J Viral Hepat.

[12] Chon YE, Kim SU, Lee CK, Heo J, Kim JK, Yoon KT (2011). Partial virological response to entecavir in treatment-naive patients with chronic hepatitis B. Antivir Ther.

[13] Guo XM, Carroll JWN, MacDonald MR, Goff SP, Gao GX (2004). The zinc finger antiviral protein directly binds to specific viral mRNAs through the CCCH zinc finger motifs. J Virol.

[14] Bick MJ, Carroll JW, Gao G, Goff SP, Rice CM, MacDonald MR (2003). Expression of the zinc-finger antiviral protein inhibits alphavirus replication. J Virol.

[15] Muller S, Moller P, Bick MJ, Wurr S, Becker S, Gunther S (2007). Inhibition of filovirus replication by the zinc finger antiviral protein. J Virol.

[16] Wang X, Tu F, Zhu Y, Gao G (2012). Zinc-finger antiviral protein inhibits XMRV infection. PLoS One.

[17] Zhu Y, Chen G, Lv F, Wang X, Ji X, Xu Y (2011). Zinc-finger antiviral protein inhibits HIV-1 infection by selectively targeting multiply spliced viral mRNAs for degradation. Proc Natl Acad Sci U S A.

[18] Mao R, Nie H, Cai D, Zhang J, Liu H, Yan R (2013). Inhibition of hepatitis B virus replication by the host zinc finger antiviral protein. PLoS Pathog.

[19] MacDonald MR, Machlin ES, Albin OR, Levy DE (2007). The zinc finger antiviral protein acts synergistically with an interferon-induced factor for maximal activity against alphaviruses. J Virol.

[20] Tang H, McLachlan A (2002). A pregenomic RNA sequence adjacent to DR1 and complementary to epsilon influences hepatitis B virus replication efficiency. Virology.

[21] Tang H, McLachlan A (2001). Transcriptional regulation of hepatitis B virus by nuclear hormone receptors is a critical determinant of viral tropism. Proc Natl Acad Sci U S A.

[22] Liu F, Song Y, Liu D (1999). Hydrodynamics-based transfection in animals by systemic administration of plasmid DNA. Gene Ther.

[23] Tang H, Delgermaa L, Huang F, Oishi N, Liu L, He F (2005). The transcriptional transactivation function of HBx protein is important for its augmentation role in hepatitis B virus replication. J Virol.

[24] Chen S, Xu Y, Zhang K, Wang X, Sun J, Gao G (2012). Structure of N-terminal domain of ZAP indicates how a zinc-finger protein recognizes complex RNA. Nat Struct Mol Biol.

[25] Gong DY, Chen EQ, Huang FJ, Leng XH, Cheng X, Tang H (2013). Role and functional domain of hepatitis B virus X protein in regulating HBV transcription and replication in vitro and in vivo. Viruses.

[26] Ye P, Liu S, Zhu Y, Chen G, Gao G (2010). DEXH-Box protein DHX30 is required for optimal function of the zinc-finger antiviral protein. Protein Cell.

[27] Guo X, Ma J, Sun J, Gao G (2007). The zinc-finger antiviral protein recruits the RNA processing exosome to degrade the target mRNA. Proc Natl Acad Sci U S A.

[28] Gao G, Guo X, Goff SP (2002). Inhibition of retroviral RNA production by ZAP, a CCCH-type zinc finger protein. Science.

[29] Liu FJ, Liu L, He F, Wang S, Zhou TY, Liu C (2007). Establishment and primary application of a mouse model with hepatitis B virus replication. World J Gastroenterol.

[30] He F, Chen EQ, Liu L, Zhou TY, Liu C, Cheng X (2012). Inhibition of hepatitis B Virus replication by hepatocyte nuclear factor 4-alpha specific short hairpin RNA. Liver Int.

[31] Liu FJ, Chen EQ, Zhou QL, Zhou TY, Liu C, Liu L (2012). Functional characterization of interferon regulation element of hepatitis B virus genome In Vivo. Indian J Virol.

[32] Tang H, Banks KE, Anderson AL, McLachlan A (2001). Hepatitis B virus transcription and replication. Drug News Perspect.

